# Geranylgeranylacetone reduces cardiomyocyte stiffness and attenuates diastolic dysfunction in a rat model of cardiometabolic syndrome

**DOI:** 10.14814/phy2.15788

**Published:** 2023-11-20

**Authors:** Mark T. Waddingham, Vasco Sequeira, Diederik W. D. Kuster, Elisa Dal Canto, M. Louis Handoko, Frances S. de Man, Denielli da Silva Gonçalves Bós, Coen A. Ottenheijm, Shengyi Shen, Robbert J. van der Pijl, Jolanda van der Velden, Walter J. Paulus, Etto C. Eringa

**Affiliations:** ^1^ Department of Physiology, Amsterdam Cardiovascular Sciences Amsterdam University Medical Centers Amsterdam The Netherlands; ^2^ Department of Cardiac Physiology National Cerebral and Cardiovascular Center Suita Japan; ^3^ Laboratory of Experimental Cardiology University Medical Center Utrecht Utrecht The Netherlands; ^4^ Julius Center for Health Sciences and Primary Care University Medical Center Utrecht Utrecht The Netherlands; ^5^ Department of Cardiology, Amsterdam Cardiovascular Sciences Amsterdam University Medical Centers Amsterdam The Netherlands; ^6^ Department of Pulmonology, Amsterdam Cardiovascular Sciences Amsterdam University Medical Centers Amsterdam The Netherlands; ^7^ Cellular and Molecular Medicine University of Arizona Tucson Arizona USA

**Keywords:** cardiomyocyte, diastolic heart failure, ejection fraction, myofilament protein, small heat shock proteins

## Abstract

Titin‐dependent stiffening of cardiomyocytes is a significant contributor to left ventricular (LV) diastolic dysfunction in heart failure with preserved LV ejection fraction (HFpEF). Small heat shock proteins (HSPs), such as HSPB5 and HSPB1, protect titin and administration of HSPB5 in vitro lowers cardiomyocyte stiffness in pressure‐overload hypertrophy. In humans, oral treatment with geranylgeranylacetone (GGA) increases myocardial HSP expression, but the functional implications are unknown. Our objective was to investigate whether oral GGA treatment lowers cardiomyocyte stiffness and attenuates LV diastolic dysfunction in a rat model of the cardiometabolic syndrome. Twenty‐one‐week‐old male lean (*n* = 10) and obese (*n* = 20) ZSF1 rats were studied, and obese rats were randomized to receive GGA (200 mg/kg/day) or vehicle by oral gavage for 4 weeks. Echocardiography and cardiac catheterization were performed before sacrifice at 25 weeks of age. Titin‐based stiffness (F_passive_) was determined by force measurements in relaxing solution with 100 nM [Ca^2+^] in permeabilized cardiomyocytes at sarcomere lengths (SL) ranging from 1.8 to 2.4 μm. In obese ZSF1 rats, GGA reduced isovolumic relaxation time of the LV without affecting blood pressure, EF or LV weight. In cardiomyocytes, GGA increased myofilament‐bound HSPB5 and HSPB1 expression. Vehicle‐treated obese rats exhibited higher cardiomyocyte stiffness at all SLs compared to lean rats, while GGA reduced stiffness at SL 2.0 μm. In obese ZSF1 rats, oral GGA treatment improves cardiomyocyte stiffness by increasing myofilament‐bound HSPB1 and HSPB5. GGA could represent a potential novel therapy for the early stage of diastolic dysfunction in the cardiometabolic syndrome.

## 1 INTRODUCTION

Heart failure (HF) is a significant public health issue, and it is the primary cause of hospitalization in Western countries (Dickstein et al., 2008). HF is classified based on ejection fraction (EF), which is a simple measure of the global systolic function of the left ventricle (LV) (Ponikowski et al., 2016; Yancy Clyde et al., 2013). According to guidelines from North American and European cardiology societies, HF is categorized into HF with reduced EF (HFrEF), HF with mid‐range EF (HFmrEF) and HF with preserved EF (HFpEF), depending on whether the LVEF is ≤40%, ≥41%–≤49% or ≥50%, respectively (Ponikowski et al., 2016; Savarese et al., 2022; Yancy Clyde et al., 2013). HFrEF is caused by myocardial damage such as myocardial ischemia or myocarditis, resulting in loss of contractile function (Brouwers et al., 2013; González et al., 2011). HFpEF is a multi‐organ syndrome with high heterogeneity and phenotypic complexity, and numerous comorbidities such as obesity, diabetes, and arterial hypertension critically contribute to its pathophysiology. The cluster of metabolic disorders that increase the risk of HFpEF (Packer, 2020), as well as other cardiovascular diseases, is referred to as the cardiometabolic syndrome (von Bibra et al., 2016). The defining features of HFpEF are abnormal LV compliance and elevated filling pressures, which distinguish it from HFmrEF and HFrEF (Paulus & Tschöpe, 2013). Although recent clinical trials have shown beneficial effects of treatments of SGLT2 inhibitors on symptoms in HFpEF (Anker et al., 2021; Solomon et al., 2022), improvement of myocardial diastolic dysfunction associated with HFpEF remains an unmet clinical need. This highlights the need for a deeper understanding and targeting of the pathomechanisms underlying myocardial stiffening in HFpEF (Dunlay et al., 2017).

The past two decades of research have revealed several critical pathomechanisms contributing to myocardial remodeling in HFpEF, including cytoskeletal and myofilament protein‐mediated viscoelastic remodeling in response to persistent exposure to metabolic and hemodynamic stressors (Hamdani & Paulus, 2013; van Heerebeek, Franssen, et al., 2012). Increased viscoelastic remodeling (lower compliance) of the myocardium is observed in human HFpEF (Hamdani & Paulus, 2013; van Heerebeek, Franssen, et al., 2012). Examination of LV endomyocardial biopsies shows a large proportion of HFpEF patients have only minor LV fibrosis (Mohammed et al., 2015) and high titin‐based cardiomyocyte stiffness have been shown to be an important contributing mechanism (Borbély et al., 2005). Changes in titin compliance in HFpEF are primarily attributed to posttranslational modulation via hypo‐ or hyperphosphorylation of the titin molecule, driven by inflammation‐induced reductions in nitric oxide (NO) availability (Hamdani & Paulus, 2013). This leads to a decrease in NO‐stimulated cyclic guanosine monophosphate (cGMP) levels and diminished cardiomyocyte protein kinase G (PKG) activity (Paulus & Tschöpe, 2013). Indeed, preclinical studies have demonstrated that PKG administration lowers cardiomyocyte stiffness as well as elevated titin‐based stiffness of HFpEF cardiomyocytes (Franssen et al., 2016; van Heerebeek, Hamdani, et al., 2012). Due to the direct contribution of the function of individual cardiomyocytes to the in vivo performance of the heart, diastolic dysfunction in HFpEF and the cardiometabolic syndrome are thought to be caused by defective NO‐cGMP‐PKG signaling (Franssen et al., 2016; Schiattarella et al., 2019; van Heerebeek, Hamdani, et al., 2012). However, attempts to improve diastolic function by increasing PKG activity with sildenafil (Redfield et al., 2013), vericiguat (Redfield et al., 2015), and organic and inorganic nitrates (Borlaug et al., 2018; Pieske et al., 2017), have all failed in the clinical setting.

Geranylgeranylacetone (GGA), a related isoprenoid compound to geranylgeranylpyrophosphate (Hashimoto et al., 2005), is a pharmacological drug that has been studied as a possible treatment for a variety of conditions such as ulcers, cardiomyopathy, and neurodegenerative diseases (Brundel et al., 2006; Keiji et al., 1998; van Marion et al., 2020). GGA has been used to treat gastric ulcers and gastritis since 1984 (Keiji et al., 1998), to protect dogs from developing atrial fibrillation through induction of HSPB1 (Brundel et al., 2006; van Marion et al., 2020), and to preserve post‐ischemic myocardial contractility (Marunouchi et al., 2014). Short‐term oral GGA therapy in humans has been shown to raise HSPB1 expression in the myocardium (van Marion et al., 2020) and increase microvascular NO activity in muscle (Fujimura et al., 2012). In addition to expression, GGA influences intracellular protein trafficking and cytoskeletal structure by changing protein prenylation (Hashimoto et al., 2005). Prenylation is an important mechanism for regulation of heat shock proteins (Hála & Žárský, 2019).

Because small HSPs can lower viscoelastic stiffness in failing human cardiomyocytes (Franssen et al., 2017), we investigated the effects of oral GGA treatment on stiffness of isolated permeabilized cardiomyocytes and global cardiac function in the obese Zucker fatty/spontaneously hypertensive F1 (ZSF1) rat. The ZSF1 rat has been widely studied as an experimental model for cardiometabolic syndrome (Abdellatif et al., 2021), with hypertensive rats being either heterozygous or homozygous for leptin receptor deficiency, resulting in a lean or obese phenotype, respectively (Franssen et al., 2016; Hamdani et al., 2013; van Dijk Christian et al., 2016). Obese ZSF1 rats initially develop declining renal function and LV hypertrophy along with impaired diastolic relaxation and eventually, pulmonary congestion (Franssen et al., 2016; Hamdani et al., 2013; van Dijk Christian et al., 2016). Previous studies demonstrated that elevated titin‐based cardiomyocyte stiffness precede any significant LV hemodynamic changes in 20‐week‐old obese ZSF1 rats (Franssen et al., 2016; Hamdani et al., 2013; van Dijk Christian et al., 2016). This provides a unique opportunity to investigate potential improvements to cardiomyocyte relaxation and cellular diastolic function, at an early stage of HFpEF development.

In this study, obese 21‐week‐old ZSF1 rats were administered GGA orally for four weeks prior to sacrifice. The cardiomyocytes of treated, obese ZSF1 rats were then compared to those of 25‐week‐old untreated, obese ZSF1 rats and 25‐week‐old untreated, lean ZSF1 rats. Our data show that oral delivery of GGA significantly improves cardiomyocyte compliance in association with upregulation of myofilament‐bound HSPB1 and HSPB5. As such GGA is a promising therapeutic modality for HFpEF, adding a much‐needed treatment option to the current therapeutic approaches.

## 2 METHODS

### 2.1 Animals and study design

Eight‐week‐old, male lean (*n* = 10) and obese (*n* = 20) ZSF1 rats were sourced from Charles River Laboratories (MA, USA). Rats were housed in a controlled environment and provided access to a standard laboratory rodent diet and water ad libitum. At 21 weeks of age, obese ZSF1 rats were randomized to receive GGA (200 mg/kg/day; Eisai, Tokyo, Japan) or the equivalent volume of vehicle solution (5 mL/kg) via oral gavage and were followed for a further 4 weeks. Lean rats received only the vehicle solution. Body weight was measured biweekly, and determination of blood glucose concentrations was performed in the fasted state (~12–14 h overnight) at 25 weeks of age in all rats.

### 2.2 Echocardiography

Echocardiographic assessment of cardiac function was performed at 21 and 25 weeks of age as previously described (Handoko et al., 2009). In brief, rats were anesthetized with 4% isoflurane (1:1 mixture with O_2_ and Air; Pharmachemie, Haarlem, The Netherlands) and maintained free‐breathing with 3% isoflurane. Transthoracic echocardiography was performed with a ProSound SSD‐4000 system equipped with a 13‐MHz linear transducer (UST‐5542, Aloka, Tokyo, Japan). Analyses were performed off‐line (Image‐Arena 2.9.1, TomTec Imaging Systems, Unterschleissheim, Munich, Germany). LV end‐diastolic, end‐systolic and stroke volumes were calculated from linear measurements assuming a spherical shape of the LV and then used to calculate LV ejection fraction (LVEF) and LV mass (LVM). Aortic flow velocity was recorded by pulsed‐wave Doppler above the aortic valve. To obtain cardiac output (CO), stroke volume was multiplied by heart rate. For evaluation of diastolic function, mitral valve deceleration time (DT) was measured with pulsed‐wave Doppler above the mitral leaflets. Isovolumetric relaxation time (IVRT) was measured in apical long‐axis view by placing the sample volume in the LV outflow tract with continuous‐wave Doppler. Left atrial anteroposterior dimensions and area (LAA) were measured at their maximum, by 2D echocardiography in the parasternal long‐axis and in the four‐chamber view respectively.

### 2.3 Invasive left ventricular catheterization

Left ventricular catheterization was performed as described (da Silva Gonçalves Bós et al., 2018). Immediately after the 25‐week echocardiography, invasive left ventricular catheterization was performed. Rats were intubated with a 16G catheter (Angiocath, Becton Dickinson, NJ, USA) and artificially ventilated with isoflurane (2%–3% 1:1 mixture with O_2_ and Air) at ~75 breaths/min with a tidal volume of 6 mL/kg. Body temperature was maintained at 36–37°C with the use of a heat lamp. Once deep surgical anesthesia was confirmed by the absence of the pedal reflex, rats underwent a full thoracotomy to expose the heart for catheterization. A 2Fr miniaturized combined, catheter‐micromanometer (model SPR‐838; Millar Instruments, TX, USA) was then inserted into the LV along its long‐axis using the apical stab method (Pacher et al., 2008). A 1.4Fr micromanometer (model SPR‐671, Millar Instruments) was also inserted into the right common carotid artery for the continuous measurement of arterial blood pressure. The inferior vena cava (IVC) was then isolated and a loose ligature placed around the vessel to facilitate cardiac preload reduction. Rats were allowed a stabilization period (~10–15 min) after the surgical preparation before pressure‐volume (PV) loop recordings commenced.

PV loops were recorded at steady‐state and then during preload reduction by a brief occlusion of the IVC. All recordings were made with the rat apneic by briefly suspending ventilation (~5–10 s). PV loop and arterial blood pressure data were acquired using an MPVS‐300 (Millar Instruments) connected to a ML880 PowerLab 16/30 (ADInstruments, NSW, Australia) and recorded in CHART software (v.5.5.6, ADInstruments) at a sampling rate of 1000 Hz. PV‐loop analysis was conducted off‐line using LabChart (v7.0, ADInstruments) and PVAN (v3.6, Millar Instruments).

### 2.4 Tissue collection

At the conclusion of the experiments, rats were killed by exsanguination under isoflurane anesthesia and the heart and lungs rapidly excised. The left ventricle and interventricular septum (IVS) were weighed and transversely sliced at the level of the mid‐wall. The apical portion was minced and snap‐frozen in liquid nitrogen and stored at −80°C and the remainder fixed in 4% paraformaldehyde and routinely processed for immunofluorescence studies. Total lung weight (LW) was measured. Tibia lengths (TL) were also measured in rats and used for the normalization of organ weights, to account for the large differences in body weight between the groups.

### 2.5 Cardiomyocyte mechanics in vitro

Measurements of F_passive_ were performed in single, freshly membrane‐permeabilized cardiomyocytes as previously described (Hamdani et al., 2013). All cardiomyocyte F_passive_ measurements were conducted relaxing solution with 100 nM [Ca^2+^] at 20°C. Force values were normalized to the cardiomyocyte cross‐sectional area and reported as kN/m^2^. Sarcomere length (SL) was determined using spatial Fourier transformation. In a first set of experiments, baseline F_passive_ was measured. Permeabilized cardiomyocytes were adjusted to a SL of 1.8 μm and after developed force had stabilized, F_passive_ was determined by shortening cardiomyocytes by 20% of their length. Cardiomyocytes were then progressively adjusted to a SL of 2.0, 2.2, and 2.4 μm with the protocol repeated at each SL. In a second set of experiments, permeabilized cardiomyocytes were incubated either in recombinant human HSPB5 (0.01 mg/mL; ab48779, Abcam, Cambridge, UK) or recombinant human HSPB1 (0.01 mg/mL; ab48740, Abcam, Cambridge, UK) for 40 min at 20°C (Franssen et al., 2017; Kötter et al., 2014). Permeabilized cardiomyocytes were then moved to relaxing solution and adjusted to a SL of 1.8 μm. F_passive_ was then measured at 1.8, 2.0, 2.2, and 2.4 μm as per Baseline protocol. Finally, in a third set of experiments, effects of protein kinase A (PKA) were investigated in permeabilized cardiomyocytes at an optimal SL of 2.2 μm (Borbély et al., 2005, 2009). The basal F_passive_ of permeabilized cardiomyocytes was measured in relaxing solution at 20°C, and cardiomyocytes were then transferred into a bath containing 100 μL of exogenous PKA (1unit/μL; P5511, Sigma, MO, USA) and 0.006 mM of 3′,5′‐cyclic adenosine monophosphate (cAMP; A3262, Sigma, MO, USA) in relaxing solution at 20°C. F_passive_ was subsequently measured after transfer to the PKA/cAMP solution. The basal measurement served as the 0 min recording.

### 2.6 Tissue preparation for SDS‐PAGE and Western Blotting

Subcellular fractionation of LV + IVS myocardial tissue to the cytosolic (soluble) and the myofilament (insoluble) components were conducted according to a previously published protocol (Kuster et al., 2015). Approximately 100 mg of LV + IVS tissue was homogenized in ice‐cold F60 buffer (60 mM KCl, 30 mM Imidazole, 2 mM MgCl_2_, pH 7.4) containing protease (complete™ Mini cocktail, Roche, Basel, Switzerland) and phosphatase inhibitors (PhosSTOP, Roche, Basel, Switzerland). Homogenates were then centrifuged at ~14,000 g at 4°C, and the supernatant was collected as the cytosolic (soluble) fraction. The pellet was resuspended in ~1 mL of F60 buffer containing 1% Triton X‐100 (Sigma‐Aldrich, Darmstadt, Germany) and homogenized, centrifuged at ~14,000 g at 4°C and the supernatant discarded. The remaining pellet was resuspended in ~350 μL of RIPA buffer (Sigma‐Aldrich, Darmstadt, Germany), and this was taken as the myofilament (insoluble) fraction. After the addition of 4xLDS sample buffer and DTT and vortexing thoroughly, cytosolic and myofilament fractions were stored at −80°C. The relative amount of sHSPs associated with the myofilament evaluated as the ratio of the expression of HSPB5 or HSPB1 in the myofilament fraction over the respective expression of HSPB5 or HSPB1 in cytosolic fraction. The protein concentration of each sample was estimated using the Pierce™ 660 nm Protein Assay (Thermo Fischer Scientific, MA, USA) using a 96‐well microplate according the manufacturer's instructions.

### 2.7 SDS‐PAGE and Western blotting

10 μg of each sample was loaded on 4%–15% gradient gels (Mini or Criterion TGX™, Bio‐Rad, CA, USA) and subjected to SDS‐PAGE under reducing conditions. Following SDS‐PAGE, proteins were transferred onto PVDF membranes using a semi‐dry technique (TransBlot Turbo™, Bio‐Rad, CA, USA). After transfer, membranes were briefly rinsed in dH_2_O and stained using Ponceau Red (Sigma‐Aldrich, Darmstadt, Germany) to confirm protein transfer. Membranes were blocked in a solution of 3% BSA w/v diluted in 1xTBST for 1 h at room temperature and then incubated in primary antibodies against HSPB5 or HSP25 (rodent HSPB1) (both 1:2000 diluted with 3% BSA w/v in 1xTBST; Enzo Life Sciences, NY, USA), at 4°C overnight. The next day, membranes were washed 3 × 10 min in 1xTBST before being incubated in a goat anti‐rabbit secondary antibody (1:2500 diluted with 3% BSA w/v in 1xTBST; Dako, Glostrup, Denmark) for 1 h at room temperature. After washing in 1xTBST for 3 × 5 min, membranes were incubated in a 1:1 mixture of Enhanced Chemiluminescence (ECL) Solution A & Solution B (ECL Prime; GE Healthcare, Buckinghamshire, UK) and then imaged using an Amsersham Imager 600 (GE Healthcare). Membranes were then re‐probed with respective loading control antibodies GAPDH (cytosolic; 1:5000, Cell Signaling Technologies, MA, USA) or α‐Actinin (myofilament; 1:1500, Sigma‐Aldrich, Darmstadt, Germany) for 1 h at room temperature and then the protocol repeated as stated above.

#### 2.7.1 Titin isoform separation

Titin isoforms in LV tissue were separated on 1% agarose gels (SDS‐PAGE), and expression was analyzed as previously described (van der Pijl et al., 2020). In short, small pieces of LV + IVS tissue were solubilized in a urea buffer (8 M urea, 2 M thiourea, 0.05 M Tris–HCl [pH 6.8], 75 mM DTT, 3% SDS and 0.05% bromophenol blue), heated at 60°C for 10 min and run in a SE600X vertical gel system (Hoefer Inc, MA, USA) at 15 mA for ~3 h. Bands were visualized by staining with Neuhoff's Coomassie, scanned and then analyzed with One‐D scan EX (Scanalytics, WI, USA). The integrated optical density of titin bands was determined as a function of the slope of the linear range between the integrated optical density and the loaded volume.

### 2.8 Immunofluorescence

Sections of 4 μm were dewaxed in two changes of xylene for 5 min each and rehydrated to water through graded ethanols (2 × 100%, 1 × 80%, 1 × 50%) for 5 min each. Heat‐mediated antigen retrieval was performed by incubating sections in a citrate buffer at 98°C for 20 min with the use of a water bath. After cooling to room temperature, sections were washed in several changes of 1xPBS and blocked with 5% normal goat serum for 1 h at room temperature. Following that, sections were immunostained with a rabbit polyclonal anti‐HSPB5 or rabbit polyclonal anti‐HSP25 (rodent HSPB1) antibody (both Enzo Life Sciences, NY, USA) diluted 1:250 in PBS with 1% BSA (immunohistochemical grade; Vector Laboratories) at 4°C overnight. Anti‐rabbit conjugated to Abberior STAR 580 (Abberior GmbH) was used to visualize HSPB5 and HSP25. The membranes were stained with Wheat‐Germ Agglutination (WGA) conjugated with Alexa 488 (Thermofisher) diluted 1:100 in PBS, and the nuclei were stained with DAPI (Thermofisher) diluted 1:500 in PBS. The secondary antibody, WGA and DAPI were incubated for 2 h at room temperature. The samples were washed 3x with PBS and mounted in Mowiol mounting medium +2.5% v/v DABCO (Thermofisher). The staining was visualized with a Leica TCS SP8 3X (Leica Microsystems). DAPI, Alexa 488, and Abberior 580 were irradiated with a laser at 405, 499, and 587 nm, respectively. An oil‐immersed 63x objective with a Numerical Aperture of 1.4 was used to image each section. The fluorescent signal was detected by the use of gated Hybrid Detectors. The images were analyzed, and background levels adjusted based on a negative control without primary antibody. The analyses were done with FIJI (Schindelin et al., 2012).

### 2.9 Statistical analysis

All data are expressed as mean ± SEM unless otherwise stated. Differences between the groups were assessed with a one‐way ANOVA. The effect of recombinant sHSPs and exogenous PKA on isolated, permeabilized cardiomyocytes was assessed with a two‐way ANOVA. A mixed‐model two‐way ANOVA was used to determine effects of independent variables (age, group) from the echocardiography data. A Bonferroni's post hoc test to correct for multiple comparisons was used for both one‐way and two‐way ANOVAs. All statistical analyses were performed using GraphPad Prism (v7.0, GraphPad, CA, USA) with *p* < 0.05 considered statistically significant.

## 3 RESULTS

### 3.1 General animal characteristics and organ weights

At 25 weeks of age, obese ZSF1 rats showed significantly higher body weights and body surface areas compared to the lean rats, along with fasting hyperglycemia and systolic and diastolic arterial hypertension (Table 1). Treatment with GGA did not significantly impact body weight, body surface area, fasting blood glucose concentration, or arterial pressure in obese ZSF1 rats (Table 1). Normalized LV weight was increased in obese ZSF1 rats compared to lean controls (LV + IVS weight: tibia length 29.6 ± 1.8 vs. 25.6 ± 1.3 mg/mm, *p* < 0.0001; Table 1) but was unaffected by GGA treatment (30.2 ± 1.9 mg/mm, P=NS). Lung weight corrected for tibia length was slightly increased in obese ZSF1 rats compared to lean rats (45.7 ± 6.6 vs. 40.5 ± 4.5 mg/mm, *p* = 0.07, Table 1) with similar lung wet: dry weight ratio (Table 1), suggesting early pulmonary pathology.

**TABLE 1:** General characteristics and morphometrics of lean and obese ZSF1 rats at 25 weeks of age.

	Lean (*n* = 9)	Obese (*n* = 10)	Obese + GGA (*n* = 9)
*General animal characteristics*			
Body weight (g)	474 ± 5	**668 ± 20** ^**##**^	**650 ± 18** ^**##**^
Body surface area (cm^2^)	553 ± 4	**695 ± 14** ^**##**^	**687 ± 9** ^**##**^
Fasted blood glucose concentration (mmol/L)	6.4 ± 0.4	12.5 ± 1.7**	11.4 ± 0.9*
Mean arterial pressure (mmHg)	137 ± 4	**160 ± 2** ^**#**^	155 ± 5**
*Morphometrics*			
Heart weight to tibial length ratio (mg/mm)	34.3 ± 0.6	**40.6 ± 0.8** ^**##**^	**42.0 ± 1.0** ^**##**^
LV + IVS to tibial length ratio (mg/mm)	25.6 ± 0.4	**29.6 ± 0.6** ^**##**^	**30.2 ± 0.6** ^**##**^
Lung weight to tibial length ratio (mg/mm)	40.5 ± 1.5	**45.7 ± 2.2** ^**§**^	42.3 ± 1.0
Lung wet/dry weight ratio (mg/mg)	4.8 ± 0.06	4.9 ± 0.07	4.7 ± 0.14

*Note*: Data expressed as mean ± SEM. A one‐way ANOVA was used to establish differences between the groups. *p < 0.05, **p < 0.01, ^#^ *p* < 0.001, ^##^ *p* < 0.0001 vs. Lean. ^§^ *p* = 0.07 vs. Lean. For lung wet/dry weight ratio, *n* = 4–5 rats/group.

Abbreviations: LV + IVS, left ventricle + interventricular septum.

The bolded values indicate that are statistically significantly different.

### 3.2 Cardiac function in vivo

Echocardiography at baseline (21 weeks of age) revealed that both groups of obese ZSF1 rats showed signs of volume expansion when compared to lean ZSF1 rats, manifest from larger left atrial area (LAA) and LV end‐diastolic volume (LVEDV, Table 2). Interestingly, obese ZSF1 rats also exhibited greater deceleration time at baseline compared to the lean ZSF1 rats (DT; both *p* = 0.01 vs. Lean ZSF1 rats). Both groups of obese ZSF1 rats tended to exhibit a greater stroke volume compared to lean ZSF1 rats (SV; Table 2). However, lower heart rates in obese ZSF1 rats (*p* < 0.001 for group, Table 2) resulted in similar cardiac outputs at both time points among the groups (CO; Table 2). Other measures of LV systolic function, such as fractional shortening and ejection fraction, showed no differences at either time points across the groups, although a modest reduction of both parameters with age was observed (Table 2).

**TABLE 2:** Echocardiography and left ventricular hemodynamics of lean and obese ZSF1 rats at 21‐ and 25 weeks of age.

*n*	Lean		Obese		Obese + GGA		Mixed‐model ANOVA		
	Baseline (week 21)	Final (week 25)	Baseline (week 21)	Final (week 25)	Baseline (week 21)	Final (week 25)	Age (A)	Group (G)	Interaction (A × G)
	9	9	10	10	10	9	*p*‐value	*p*‐value	*p*‐value
*Echocardiography*									
Heart rate (BPM)	357 ± 13	**313 ± 17** ^**††**^	**286 ± 9** ^**#**^	270 ± 9*	**293 ± 15** ^**#**^	275 ± 7	**<0.01**	**<0.001**	NS
CO (mL/min)	44 ± 3	37 ± 2	45 ± 5	42 ± 3	44 ± 4	40 ± 2	**<0.05**	NS	NS
SV (μL)	124 ± 10	120 ± 7	160 ± 16	160 ± 13*	151 ± 16	145 ± 11	NS	NS	NS
EDV (μL)	172 ± 13	175 ± 10	229 ± 21	241 ± 20*	216 ± 22	220 ± 16	NS	**<0.05**	NS
ESV (μL)	28 ± 4	56 ± 4	69 ± 7	81 ± 8*	65 ± 7	75 ± 6	**<0.01**	**<0.05**	NS
LAA (mm^2^)	3.1 ± 0.04	**3.5 ± 0.07** ^**†**^	**3.8 ± 0.13** ^**#**^	**4.2 ± 0.07** ^**## †**^	3.5 ± 0.12	**4.1 ± 0.14** ^**‡**^	**<0.0001**	**<0.0001**	NS
LVIDd (mm)	6.9 ± 0.2	6.9 ± 0.1	7.5 ± 0.2	7.7 ± 0.2*	7.4 ± 0.2	7.5 ± 0.2	NS	**<0.05**	NS
LVIDs (mm)	4.5 ± 0.1	4.7 ± 0.1	5.0 ± 0.2	5.3 ± 0.2*	4.9 ± 0.2	5.2 ± 0.1	**<0.0001**	**<0.01**	NS
LV mass (mg)	608 ± 36	**684 ± 26** ^**††**^	744 ± 36	857 ± 37** ^**‡‡**^	752 ± 41* ^**‡**^	834 ± 38	**<0.01**	**<0.0001**	NS
FS (%)	35 ± 0.6	**32 ± 0.6** ^**‡**^	33 ± 0.9	**31 ± 0.9** ^**‡**^	33 ± 1	**30 ± 0.8** ^**‡**^	**<0.0001**	NS	NS
EF (%)	72 ± 0.8	**68 ± 0.9** ^**††**^	70 ± 1.2	**66 ± 1.4** ^**‡**^	70 ± 1.4	**66 ± 1.1** ^**‡**^	**<0.0001**	NS	NS
IVRT (ms)	39 ± 2.3	45 ± 3.9	40 ± 2.5	**52 ± 2.5** ^**‡**^	45 ± 2.2	49 ± 2.4	**<0.0001**	NS	**<0.05**
DT (ms)	40 ± 1.8	**47 ± 1.2** ^**††**^	49 ± 2.6*	57 ± 3.0* ^**‡**^	50 ± 2.1*	53 ± 2.8	**<0.0001**	**<0.01**	NS
E/A	1.4 ± 0.05	1.7 ± 0.13	1.5 ± 0.07	1.6 ± 0.07	1.4 ± 0.06	1.6 ± 0.08	**<0.01**	NS	NS
*Invasive left ventricular hemodynamics*									
Heart rate (BPM)	–	299 ± 14	–	259 ± 10	–	276 ± 10	–	–	–
LVPSP (mmHg)	–	132 ± 9	–	153 ± 6	–	154 ± 8	–	–	–
LVEDP (mmHg)	–	4.9 ± 0.3	–	7.0 ± 1.8	–	5.9 ± 0.7	–	–	–
dP/dt_max_ (mmHg/s)	–	6823 ± 735	–	7093 ± 378	–	7376 ± 675	–	–	–
dP/dt_min_ (mmHg/s)	–	−7143 ± 777	–	−7793 ± 570	–	−8185 ± 526	–	–	–
Tau (ms)	–	10.9 ± 0.9	–	11.7 ± 1.0	–	12.9 ± 2.9	–	–	–
EDPVR β (μL^−1^)	–	0.009 ± 0.002	–	0.006 ± 0.001	–	0.006 ± 0.001	–	–	–

*Note*: Data expressed as mean ± SEM. A mixed‐model two‐way ANOVA was used to determine the effects of independent variables and a Bonferroni's post hoc test was used for comparisons between the 21‐ and 25‐week time points. A one‐way ANOVA with a Bonferroni's post hoc test was used to establish differences between the groups at week 21 and week 25 time points. ^†^ *p* < 0.05, ^††^ *p* < 0.01, ^‡^ *p* < 0.001 vs. Baseline; *p < 0.05, **p < 0.01, ^#^ *p* < 0.001, ^##^ *p* < 0.0001 vs. Lean at respective time points.

Abbreviations: CO, cardiac output; DT, deceleration time; E/A, ratio of the early (E) wave and late (A) wave during diastolic filing; EDPVR, end‐diastolic pressure volume relationship.; EDV, end‐diastolic volume; EF, ejection fraction; ESV, end systolic volume; FS, fractional shortening; IVRT, isovolumic relaxation time; LAA, left atrial area; LV, left ventricle; LVEDP, left ventricular end‐diastolic pressure; LVIDd, left ventricular internal diameter end diastole; LVIDs, left ventricular internal diameter end systole; LVPSP, left ventricular peak systolic pressure; SV, stroke volume.

The bolded values indicate that are statistically significantly different.

**FIGURE 1:**
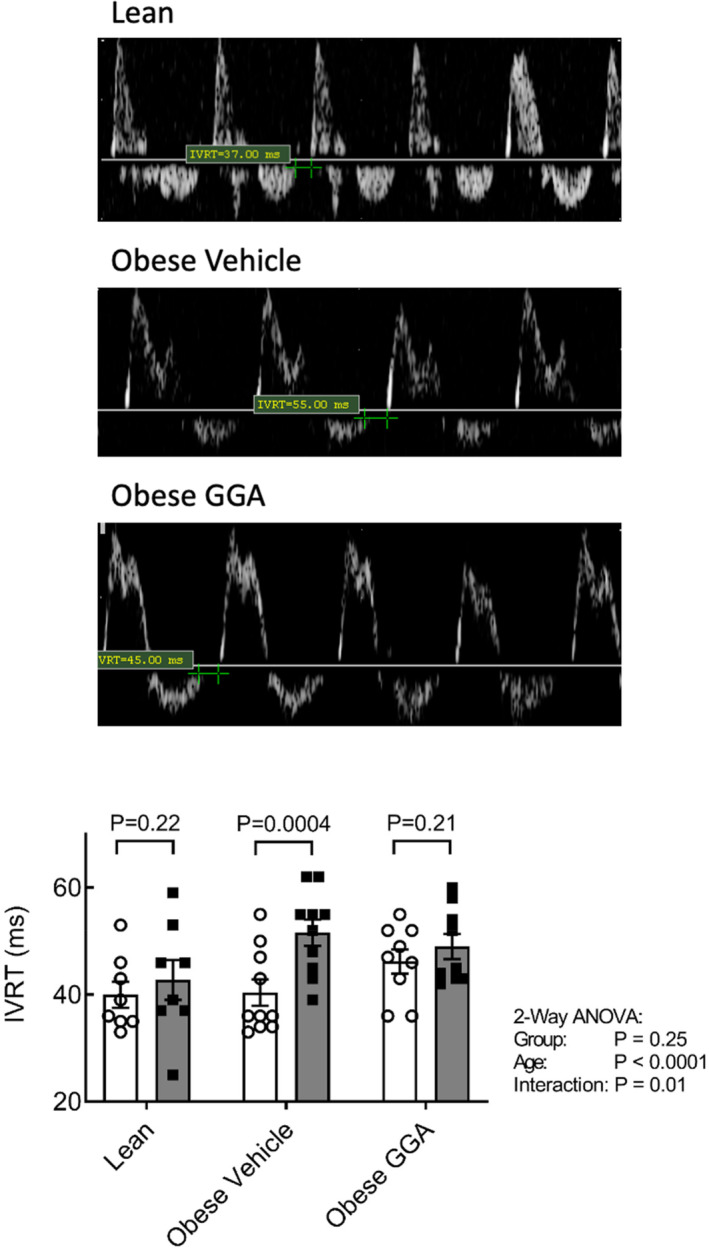
Oral GGA treatment halts the progression of diastolic dysfunction, measured as isovolumic relaxation time. Upper panel: Representative scans of transmitral pulse wave Doppler in Lean, Obese, and Obese rats treated with GGA as acquired from the apical long‐axis view at 25 weeks. Green cross‐hair cursors indicate the position used to calculate the IVRT. Lower panel: absolute IVRT values in 21‐ and 25‐week‐old lean and obese rats, measured by transthoracic echocardiography (see Methods). Effects of obesity and oral GGA treatment on progression of diastolic dysfunction, measured as the enhancement of IVRT from 21 to 25 weeks (ΔIVRT). Data are expressed as mean ± SEM. *n* = 8–10 per group at Week 21 and Week 25. A mixed‐model two‐way ANOVA was utilized to determine effects, and a Bonferroni's post hoc test was used to correct for multiple comparisons between 21‐ and 25‐week timepoints.

At the 25‐week time point, vehicle‐treated obese ZSF1 rats exhibited an average ~ 30% increase in the isovolumetric relaxation time (IVRT; Table 2) in comparison with baseline, suggesting the onset of early diastolic dysfunction. Further analysis revealed that IVRT significantly increased over time only in the vehicle‐treated obese ZSF1 rats (25 vs. 21 weeks, *p* < 0.0001; Table 2 and Figure 1). Moreover, the time‐dependent deterioration of diastolic function as reflected by the IVRT was attenuated by GGA (*p* = 0.01 vs. obese vehicle, Figure 1).

Invasive LV pressure‐volumetry assessment did not show overt diastolic impairment in obese ZSF1 rats as shown by LV end‐diastolic pressure (LVEDP), LV dP/dt_min_, the tau relaxation time constant or the end‐diastolic pressure volume relationship when compared to the lean controls (Table 2). Likewise, GGA treatment did not affect any of the diastolic indices measured using LV pressure‐volumetry (Table 2).

### 3.3 Oral GGA treatment redistributes HSPB5 and HSPB1 to the myofilaments in LV myocardium in HFpEF

**FIGURE 2:**
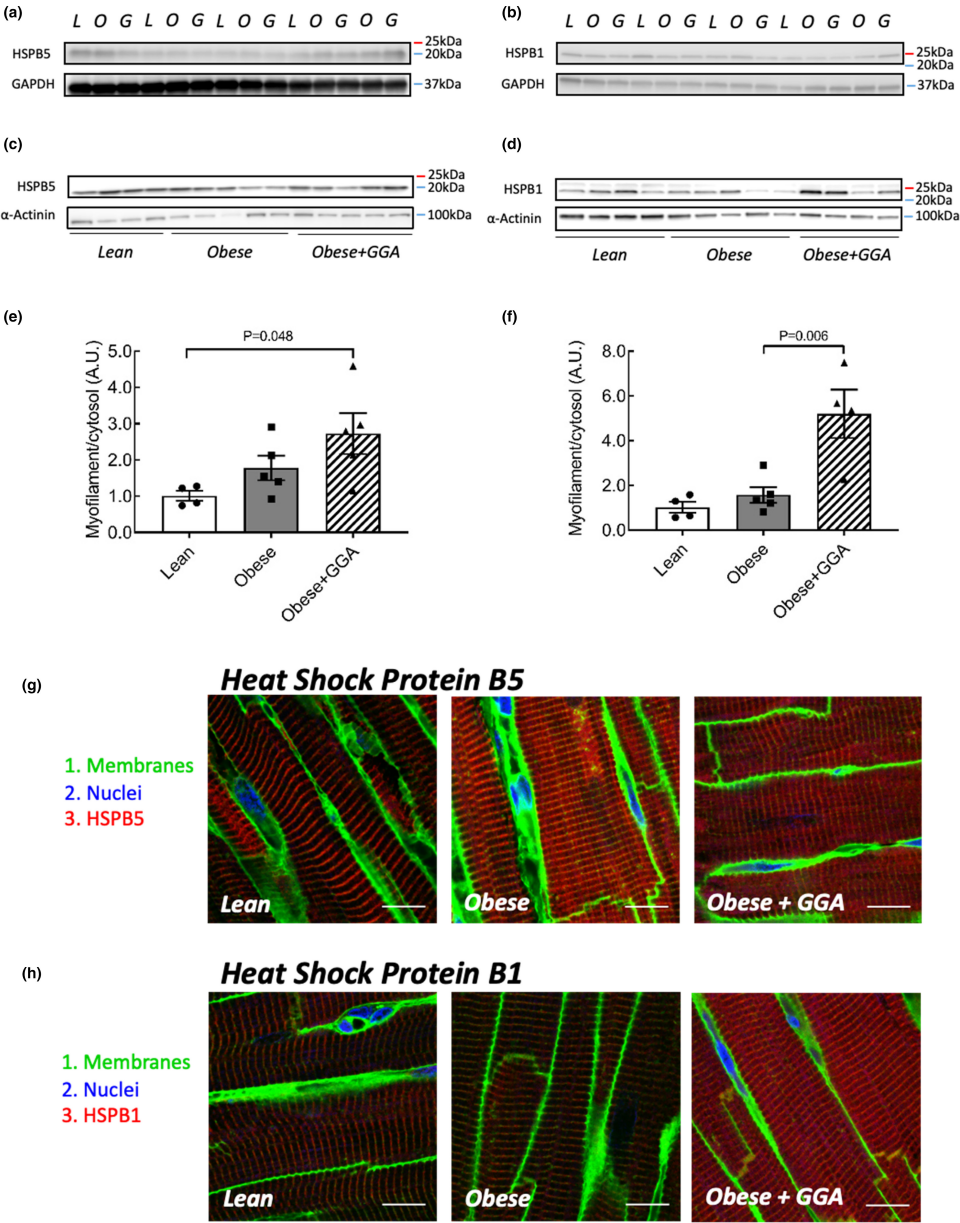
Oral GGA treatment redistributes HSPB5 and HSPB1 from the cytosol to the myofilaments in the left ventricle of obese ZSF1 rats. (a and b) HSPB5 and HSPB1 levels were similar between myocardia of all groups in cytosolic fraction. (c and d) myofilament levels of HSPB5 and HSPB1 were higher in the myocardium of obese ZSF1 rats treated with GGA when compared to vehicle‐treated obese and lean ZSF1 rats. (e and f) There was a redistribution of HSPB5 and HSPB1 to the myofilaments (myofilament expression levels relative to cytosolic expression levels) in the GGA‐treated obese ZSF1 rats compared to lean and vehicle‐treated obese ZSF1 rats, although this was particularly strong for HSPB1. Data are expressed as mean ± SEM, *n* = 4–5 per group. A one‐way ANOVA with a Bonferroni's post hoc test was utilized to assess differences between the groups. (g and h) Representative images of confocal laser microscopy for HSPB5 (upper panel) and HSPB1 (lower panel) in LV sections from lean, obese and obese ZSF1 rats treated with GGA (200 mg/kg/day). Immunohistochemical visualization of cell membranes (WGA; green), nuclei (DAPI; blue), and HSPB5/HSPB1 (red) was achieved with confocal laser microscopy. Both HSPB5 and HSPB1 immunostaining primarily occurred in the vicinity of the myofilaments for all rats. Greater HSPB5 immunostaining in the vicinity of the myofilaments was observed in both vehicle‐treated and GGA‐treated obese ZSF1 rats, whereas prominent elevation of HSPB1 immunostaining was noted in the GGA‐treated obese ZSF1 rats. *n* = 4 per group.

Following the observation of GGA‐induced attenuation of early diastolic dysfunction in obese ZSF1 rats, we conducted protein and cardiomyocyte function analyses to investigate the underlying mechanisms in cardiomyocytes. Cytosolic levels of HSPB5 in the LV myocardium were similar between groups (Figure 2a). Cytosolic HSPB5 levels relative to lean controls (1.00 ± 0.05 AU) were 1.11 ± 0.07 AU in vehicle‐treated obese rats and 1.09 ± 0.10 AU in GGA‐treated obese rats (all *p* = 1.0 vs. lean). In contrast, Cytosolic HSPB1 levels relative to lean controls (1.00 ± 0.05 AU) were lower in vehicle‐treated obese rats (0.73 ± 0.04 AU, *p* = 0.002 vs. lean) and were further decreased in GGA‐treated obese rats (0.55 ± 0.03 AU, *p* = 0.02 vs. obese vehicle). Oral GGA treatment increased myofilament‐associated HSPB5, as demonstrated by an increased ratio of myofilament HSPs over cytosolic HSPs (Figure 2c). GGA increased the myofilament/cytosolic ratio of HSPB1 by 3.3‐fold compared to vehicle‐treated obese rats (*p* = 0.006) and by 5.2‐fold compared to lean rats (*p* = 0.003; Figure 2f).

Immunofluorescence staining confirmed a primarily myofilament localization of HSPB5 and HSPB1 in the myocardium of all rats (Figures 2g,h). Notably, cardiomyocytes of GGA‐treated obese ZSF1 rats exhibited particularly strong staining of HSPB1 at the myofilament space (Figure 2g,h).

### 3.4 Passive force measurements in isolated cardiomyocytes

**FIGURE 3:**
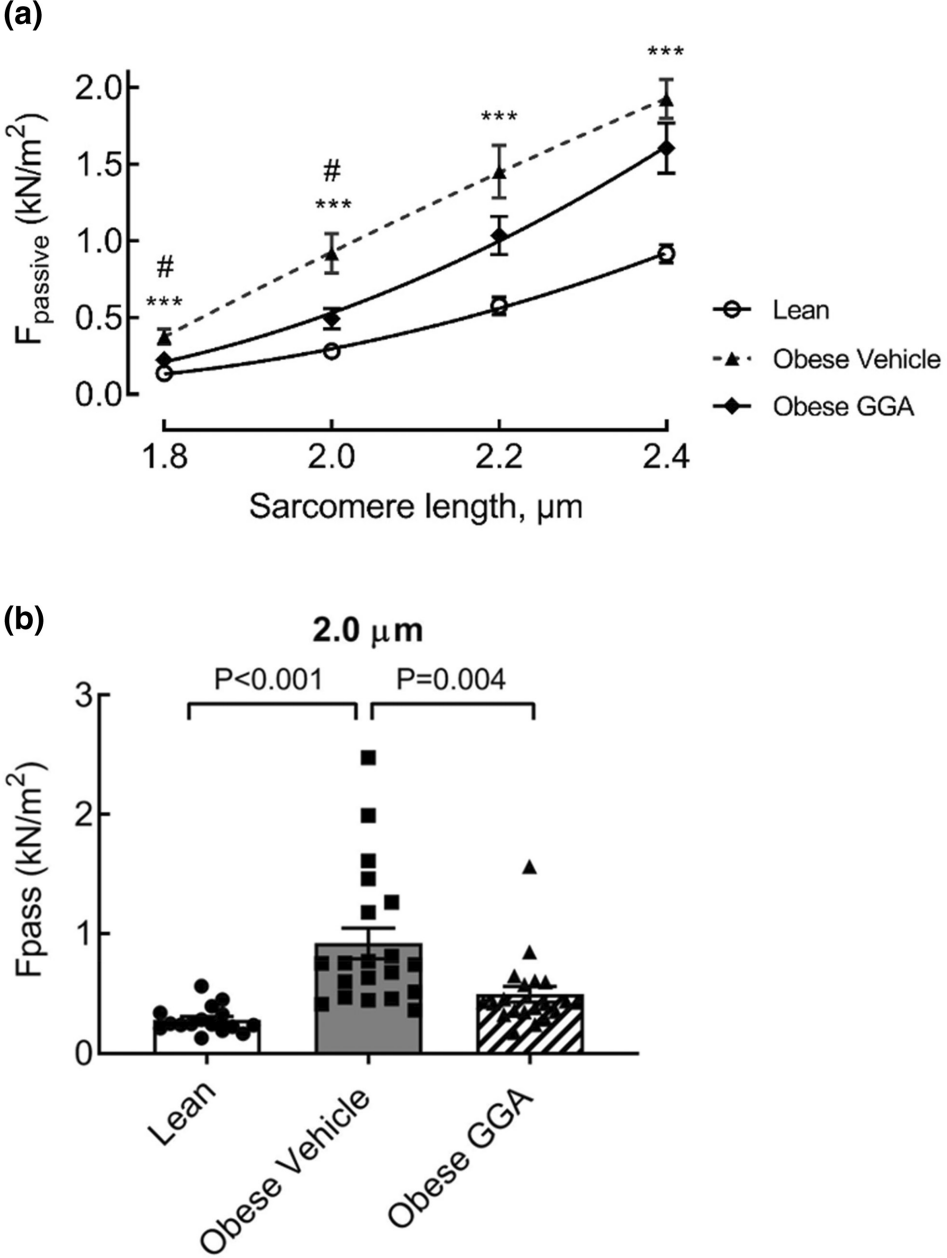
Oral GGA treatment reduces myofilament stiffness in obese ZSF1 rats. Myofilament stiffness was measured as increases in passive force (F_passive_) of isolated, permeabilized cardiomyocytes in response to increases in sarcomere length (SL). (a) SL‐F_passive_ curves for lean (solid black line; open circles), vehicle‐treated obese (dashed black line; triangles) and GGA‐treated obese (solid black line; diamonds) ZSF1 rats, using a second order polynomial (quadratic function) for curve fitting. F_passive_ was significantly higher at sarcomere length (SL) 2.0 μm in obese rats compared to lean and GGA‐treated obese rats (b). ***p < 0.001 vs. lean; ^#^ *p* = 0.02 vs. obese vehicle. Data are expressed as mean ± SD. *N* = 4–5 per group, with four cardiomyocytes tested per rat. A one‐way ANOVA with a Bonferroni's post hoc test was utilized to assess differences between the groups.

Given the close relationship between the overall cardiac function in vivo and the viscoelastic composition of individual cardiomyocytes, we conducted experiments on permeabilized cardiomyocytes as a surrogate for diastolic function at the cellular level. Cardiac muscle resists lengthening by exerting an extending force in response to stretch at nominal calcium conditions, that is, F_passive_ (Zile et al., 2015). Vehicle‐treated obese rats displayed a significant increase in passive force at all sarcomere lengths (SLs) compared to lean rats (Figure 3), resulting in an upward shift of the F_passive_‐SL relationship curve in the vehicle‐treated obese rats compared to the lean rats (Figure 3a). The latter are indicative for decreased cellular compliance (higher cardiomyocyte stiffness). Oral GGA treatment increased cardiomyocyte compliance, evidenced by lowering of F_passive_ in GGA‐treated obese ZSF1 rats at SL 2.0 μm (0.92 ± 0.13 vs. 0.49 ± 0.06 kN/m^2^, *p* < 0.01, Figure 3b) and comparable at SL 2.4 μm (2.14 ± 0.25 vs. 1.77 ± 0.23 kN/m^2^, NS, Figure 3a). The area under the full SL‐F_passive_ curve was also increased in obese rats compared to lean rats (0.73 ± 0.38 vs. 0.28 ± 0.08, *p* < 0.0001) and reduced by oral GGA treatment (to 0.51 ± 0.25, *p* = 0.025 vs. obese vehicle).

**FIGURE 4:**
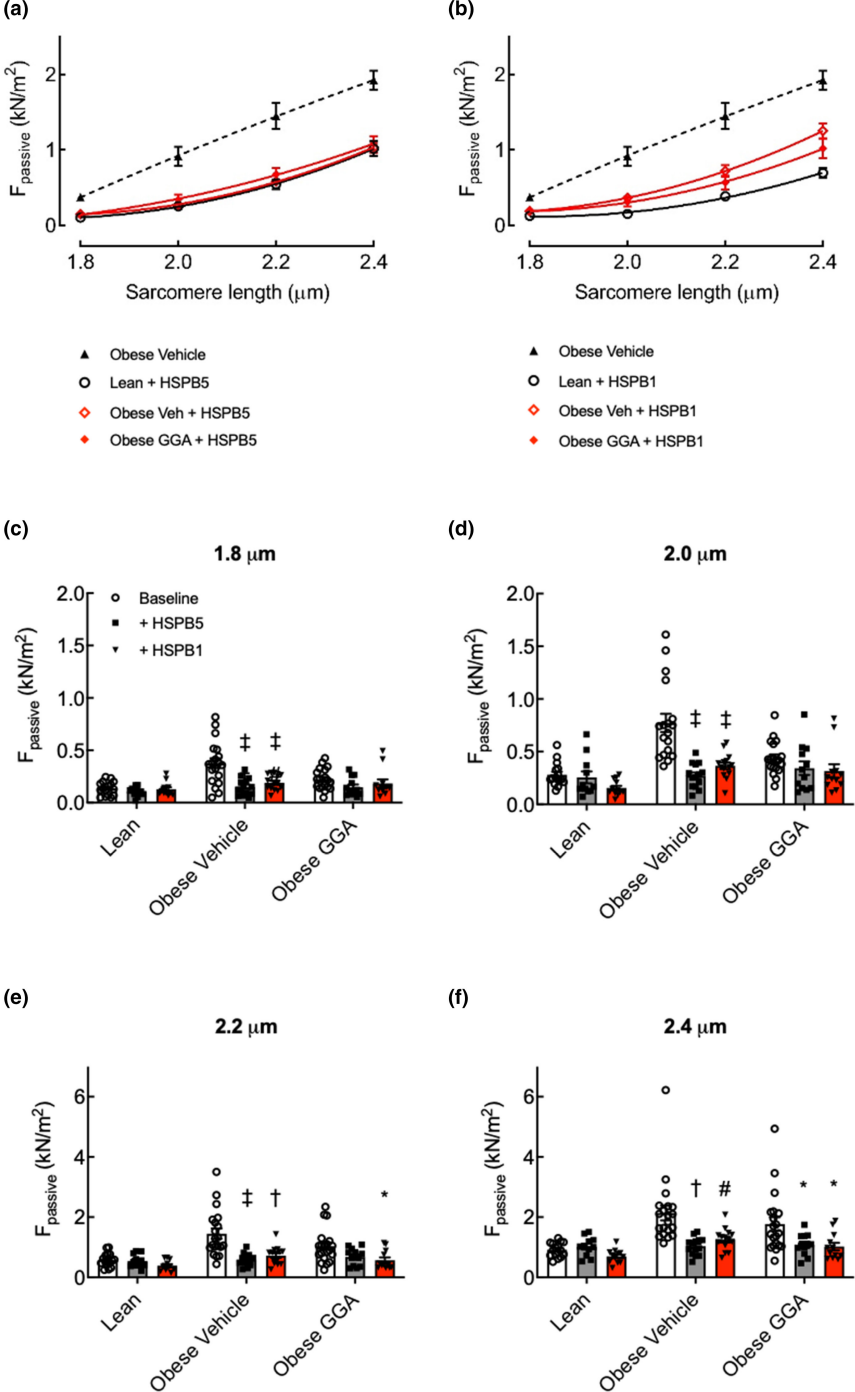
Recombinant human HSPB5 (0.01 mg/mL) and human HSPB1 (0.01 mg/mL) reduce stiffness of permeabilized cardiomyocytes of obese ZSF1 rats and abrogate the effect of oral GGA treatment. Stiffness was measured as the passive force (F_passive_). (a and b) SL‐F_passive_ curves after HSP treatment for lean, vehicle‐treated obese and GGA‐treated obese ZSF1 rats after incubation with recombinant human HSPB5 (a) or human HSPB1 (b). A second order polynomial (quadratic function) was used for curve fitting. For comparison, the F_passive_ curve of cardiomyocytes isolated from vehicle‐treated obese ZSF1 rats (dashed line, triangles) and the F_passive_ curve of cardiomyocytes isolated from lean rats are shown in panels a and b. (c–f) comparison of HSP effects on F_passive_ of cardiomyocytes in vehicle‐ and GGA‐treated rats. Data are expressed as mean ± SD. ^‡^ *p* < 0.0001, ^†^ *p* = 0.0001 and *p < 0.05 vs. baseline of the same group. *N* = 4–5 per group, with four cardiomyocytes tested per rat. Effects of recombinant sHSPs were established using a two‐way ANOVA with a Bonferroni's post hoc test to correct for differences between the groups.

Treatment of permeabilized cardiomyocytes with recombinant HSPB5 or HSPB1 abrogated the differences in F_passive_ between lean and obese rats and abolished the effect of GGA on F_passive_ in obese rats (Figure 4). In vehicle‐treated obese ZSF1 rats, treatment with HSPB5 or HSPB1 significantly reduced F_passive_ at all SLs compared to baseline values (Figures 4a,b). In contrast, in GGA‐treated obese ZSF1 rats, HSPB5 and HSPB1 reduced F_passive_ only at SLs 2.2 and 2.4 μm (Figures 4c–f). Collectively, these data suggest that raising the levels of myofilament‐associated HSPB5 and HSPB1 contributes to the effect of GGA treatment on cardiomyocyte compliance of obese ZSF1 rats.

### 3.5 Phosphorylation‐dependent and Phosphorylation‐independent changes in cardiomyocyte stiffness in obese rats

**FIGURE 5:**
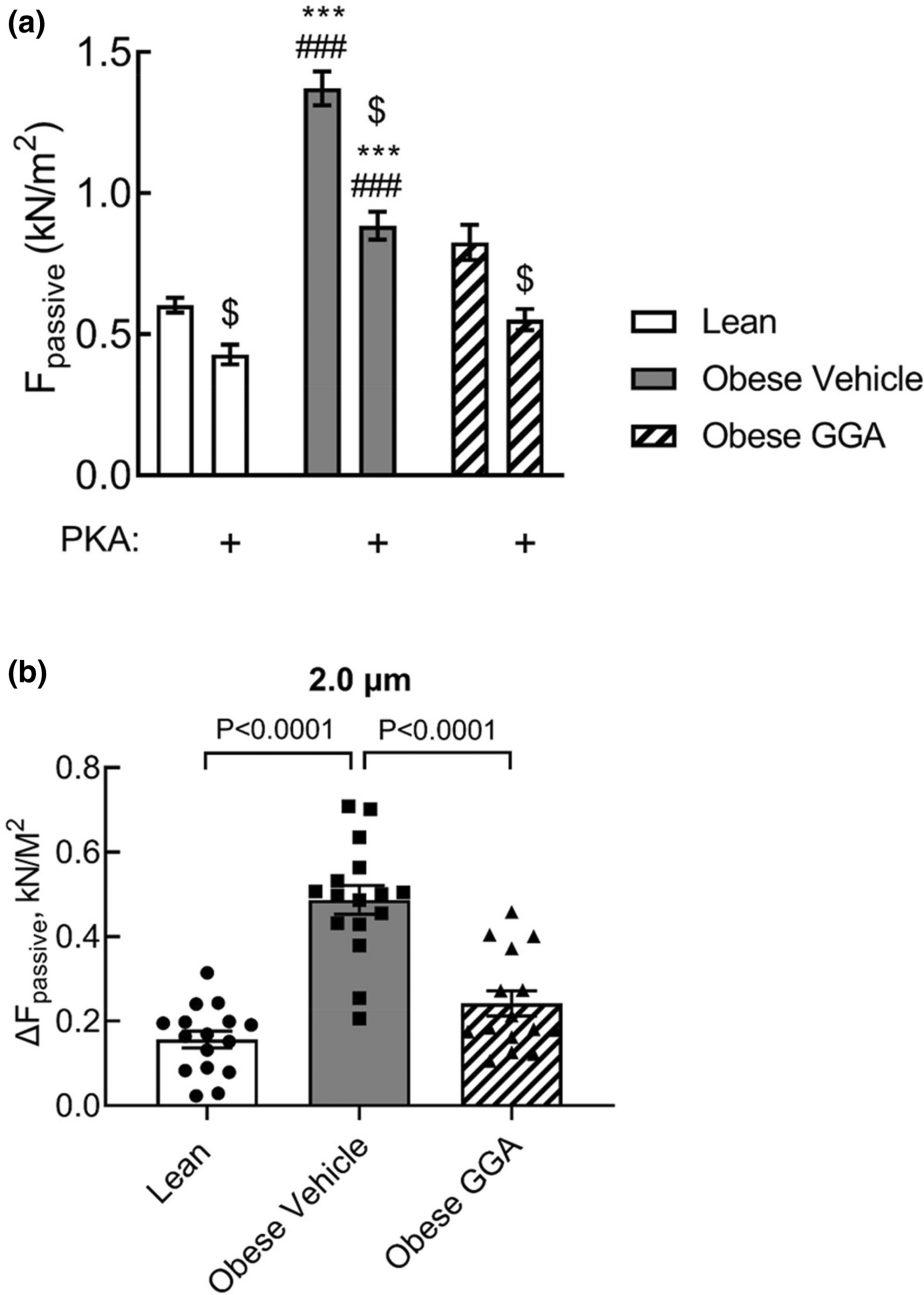
Oral GGA treatment reduces the effect of protein kinase A (PKA) on cardiomyocyte stiffness in cardiomyocytes of obese ZSF1 rats. Isolated, permeabilized cardiomyocytes of lean, obese and GGA‐treated obese rats were incubated with exogenous protein kinase A (PKA; 1 unit/μL) and 3′,5′‐cyclic adenosine monophosphate (cAMP; 0.006 mM) over a 40‐min period at an optimal sarcomere length (SL) of 2.2 μm. (a) absolute F_passive_ in the absence and presence of PKA for lean vehicle‐treated obese and GGA‐treated obese ZSF1 rats. Data are expressed as mean ± SD. *n* = 4 per group, with 4 cardiomyocytes tested per rat. ^$^ *p* < 0.0001 vs. baseline, ***p < 0.0001 vs. lean, ^###^ *p* < 0.0001 vs. Obese GGA. (b) Obesity increases the effect of PKA treatment on cardiomyocyte passive force (ΔF_passive_) compared to lean rats, and oral GGA treatment abolishes this difference. Effects of exogenous PKA was determined using a two‐way ANOVA with a Bonferroni's post hoc test to correct for differences between the groups.

To investigate the interaction between the GGA‐mediated reduction in cardiomyocyte stiffness and phosphorylation‐mediated changes in F_passive_ by incubating permeabilized cardiomyocytes with PKA. PKA‐induced phosphorylation of myofilaments is a well‐established determinant of passive force, including increased cellular compliance. PKA reduced F_passive_ at 2.2 μm SL in all groups with the largest reduction in cardiomyocytes of vehicle‐treated obese rats. However, PKA‐mediated phosphorylation in cells from obese rats untreated with GGA did not reach similar compliance levels as observed in cardiomyocytes isolated from lean ZSF1 and GGA‐treated obese animals (Figure 5a). Overall, these data illustrate that GGA reverses the increase in PKA‐enhanced cardiomyocyte compliance in obese ZSF1 rats compared to lean rats.

### 3.6 Effects of GGA on titin isoform expression

**FIGURE 6:**
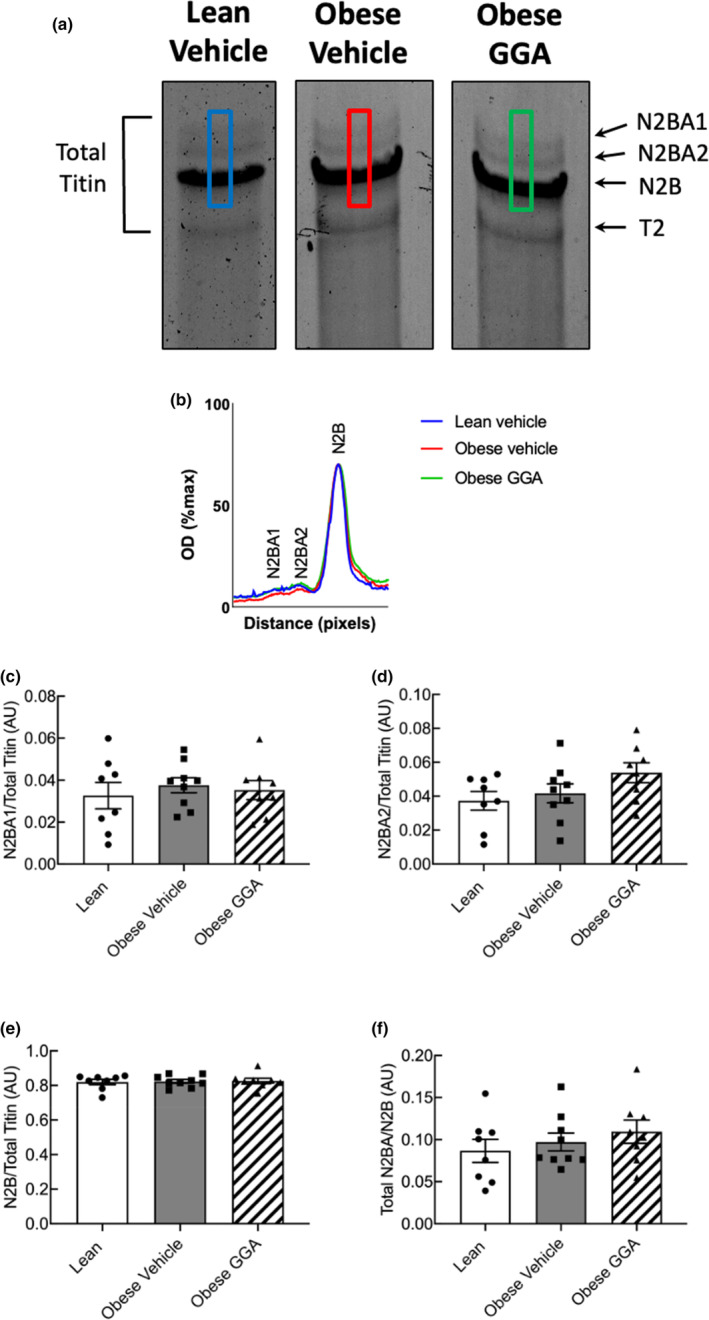
No differences in the major titin isoforms or the ratio of their expression relative to the total titin pool were observed among the groups. (a) Raw image of titin blot; (b) lane profiles for each of the raw titin blots. The analysis area is depicted by the corresponding colored rectangles in A; (c) expression of the N2BA1 isoform relative to total titin; (d) expression of the N2BA2 isoform relative to total titin; (e) expression of the N2B isoform relative to total titin; (f) the ratio between the total N2BA (N2BA1 + N2BA2) and N2B isoforms. Data are expressed as mean ± SEM. A one‐way ANOVA with a Bonferroni's post hoc test was utilized to assess differences between the groups.

As differences in passive force between lean and obese ZSF1 rats were still present in the presence of PKA, we investigated whether the beneficial effect of GGA on cardiomyocyte stiffness was linked to altered expression of titin, as changes in titin isoform expression can impact cardiomyocyte compliance. We quantified the expression of the stiff and compliant titin isoforms in lean and obese ZSF1 rats treated with either vehicle or GGA. No statistically significant differences were observed between the groups for the N2BA1 (Figure 6c), N2BA2 (Figure 6d) or the stiff N2B (Figure 6e) isoforms. Moreover, the ratio the N2BA isoforms (N2BA1 and N2BA2) and the N2B isoforms in the total titin pool was also similar between the groups (Figure 6f).

## 4 DISCUSSION

In this study, obese ZSF1 rats were used as a model for cardiometabolic syndrome, and the heat shock protein (HSP)‐inducer drug GGA was evaluated for its ability to improve numerous cardiovascular risk factors. In line with other studies (Franssen et al., 2016; Hamdani et al., 2013; van Dijk Christian et al., 2016), we show that ZSF1 rats develop LV hypertrophy with altered diastolic relaxation and increased lung weight, demonstrating pulmonary pathology (Tables 1 and 2). Given that significant changes in cardiomyocyte viscoelasticity precede major LV hemodynamic alterations in young, obese ZSF1 rats (Franssen et al., 2016; Hamdani et al., 2013; van Dijk Christian et al., 2016), we had the unique opportunity to investigate potential improvements to cardiomyocyte relaxation, a determinant of diastolic function, at an early stage in myocardial stiffening and progression toward HFpEF. Our findings indicate that four‐week treatment with GGA attenuates the progression of cardiomyocyte stiffening in obese ZSF1 rats, thereby increasing compliance at the cellular level. Mechanisms contributing to this GGA‐induced increase in cardiomyocyte compliance are 1) increased myofilament‐bound HSPB1 and HSPB5 in cardiomyocytes; and 2) lowering of the high cardiomyocyte F_passive_ characteristically observed in 25 weeks old, obese ZSF1 rats over the physiological range of sarcomere lengths (from 1.8 to 2.2 μm).

### 4.1 GGA and myofilament‐bound HSPB1 and HSPB5

The expression of total HSPB5 and HSPB1 was similar in the hearts of lean, vehicle‐treated, and GGA‐treated obese ZSF1 rats. However, GGA treatment increased HSPB5 and HSPB1 in the myofilament fraction of cardiomyocytes. The increase in myofilament‐bound HSPs induced by GGA was particularly evident for HSPB1, which showed a 2.5‐fold increase in the GGA‐treated obese ZSF1 rats. The primarily myofilament localization of both small HSPs was confirmed by immunofluorescence staining. This redistribution is functionally important, as the effect of GGA on cardiomyocyte F_passive_ was no longer present after treatment of permeabilized cardiomyocytes with exogenous recombinant HSPs (Figure 4).

In a previous study, we observed a similar association of HSPB5 with the myofilaments in LV cardiomyocytes of end‐stage dilated cardiomyopathy and symptomatic aortic stenosis patients, although their association in the present study was more pronounced (Franssen et al., 2017). Taken together, these findings demonstrate that small HSPs bind the myofilaments in human cardiomyocytes, and this binding is enhanced by GGA. The GGA‐induced redistribution of HSPs is characteristic of HSP activation (Guo et al., 2012; Snoeckx et al., 2001) and can be induced by geranylgeranylation (Hála & Žárský, 2019). As GGA is a structural homologue of the geranylgeranyl donor geranylgeranyl‐pyrophosphate (GGPP) (Hashimoto et al., 2005), which influences intracellular protein trafficking (Pronk et al., 2019), it is anticipated that GGA‐induced HSP redistribution results from protein geranylgeranylation.

Titin is a large protein found in sarcomeres that responds rapidly to physiological and pathological load by altering its compliance (Sequeira et al., 2013). As an example, in response to pressure‐overload‐induced hypertrophy, increases in myocardial viscous load via decreases in titin compliance can function as a mechanical dampener against contraction in order to reduce excessive wall stress in accordance with Laplace's law (Sequeira et al., 2013). Titin is involved in the early phase of myofilament protein‐mediated viscoelastic remodeling in HFpEF (i.e., increased viscoelasticity and lower compliance), and HSPB5 or HSPB1 binds to the titin molecule's proximal Ig and N2‐Bus segments (Kötter et al., 2014). Remodeling at these sites and subsequent coverage by small HSPs could result from excessive diastolic stretch (Franssen et al., 2017; Kötter et al., 2014) or exposure to free radicals (Bódi et al., 2017), as occurs during normal aging (Bódi et al., 2017) or caused by metabolic risk factors such as obesity or diabetes mellitus (van Heerebeek et al., 2008). In obese ZSF1 rats, excessive diastolic stretch seems unlikely because LVEDP remained low and cardiomyocytes were operating over the physiological sarcomere length range. The latter was evident from the absence of any effect of GGA treatment on cardiomyocyte F_passive_ at sarcomere lengths above the physiological sarcomere length range (>2.2 μm) and from presence at these lengths of an in vitro response to recombinant HSPB5 or HSPB1.

### 4.2 Small HSPs and cardiomyocyte stiffness

Cardiomyocyte‐based stiffness, as assessed by F_passive_, was significantly higher in vehicle‐treated, obese ZSF1 rats compared to lean ZSF1 rats at all sarcomere lengths (Figure 3). GGA treatment of obese ZSF1 rats lowered F_passive_ at a physiological sarcomere length of 2.0 μm but not at the longest (2.4 μm). This suggests that HSPB5 or HSPB1's coverage of the titin molecule is the mechanism underlying the observed decrease in cardiomyocyte stiffness due to GGA treatment. The sequential unfolding of different segments of the titin molecule determines F_passive_ upon sarcomere stretching (Trombitás et al., 1999), with tandem Ig segments mostly determining titin extensibility in the sarcomere length range from 1.8 to 2.2 μm. The N2Bus segment and segment and the PEVK segment make smaller contributions to titin extensibility in this range. However, at sarcomere lengths exceeding 2.3 μm, the PEVK and N2Bus segments become more critical determinants of titin extensibility. Coverage of titin by HSPB5 or HSPB1 probably follows the sequential unfolding of distinct segments relative to the extent of sarcomere re‐lengthening. This potentially explains why lower F_passive_ following GGA treatment was only observed at a physiological sarcomere length and not at the higher sarcomere length of 2.4 μm. It is noteworthy that both the tandem Ig and N2Bus segments of titin have binding sites for HSPB5 or HSPB1, with the former being more involved in the in vivo effect of GGA treatment on F_passive_ and the latter in the in vitro effect of recombinant HSPB1 and HSPB5 at higher sarcomere lengths (≥2.2 μm) (Kötter et al., 2014).

### 4.3 Effects of GGA on phosphorylation‐dependent and phosphorylation‐independent myofilament stiffness

The N2Bus segment of titin not only contains phosphorylation sites for the control of cardiomyocyte stiffness, but also binding sites for small HSPs (Franssen et al., 2017; Linke & Hamdani, 2014). Phosphorylation of these sites by protein kinase A (PKA) and protein kinase G (PKG) lowers cardiomyocyte stiffness by increasing the extensibility of the N2Bus segment (Krüger et al., 2009; LeWinter & Granzier, 2014). Because the HSP binding sites are located in close proximity to the PKA/PKG phosphorylation sites within the N2Bus segment, we evaluated the effect of oral GGA treatment on the in vitro effect of PKA on cardiomyocyte stiffness (Figure 5). We found that exogenous PKA exhibited a significantly more pronounced effect on lowering cardiomyocyte stiffness in vehicle‐treated obese ZSF1 cardiomyocytes compared to lean and GGA‐treated obese ZSF1 rats. The increased effect of PKA on F_passive_ in untreated obese rats could be explained by hypophosphorylation of myofilaments, which has been described in obese ZSF1 rats (Hamdani et al., 2013). The PKA‐mediated effect was reduced in GGA‐treated obese rats compared to vehicle‐treated obese rats and similar to that in lean rats, which may indicate that GGA treatment exerts an indirect effect on phosphorylation‐mediated cardiomyocyte stiffness. Nevertheless, it is important to establish the precise mechanisms by which GGA exerts its phosphorylation‐dependent effect upon titin and reducing cardiomyocyte stiffness. Future studies will be conducted to elucidate whether GGA affects myofilament and titin phosphorylation in the setting of cardiometabolic syndrome and HFpEF. Aside from phosphorylation‐dependent myofilament stiffness, GGA also reduces cardiomyocyte stiffness (Figure 5). This effect of GGA is not explained by altered expression of titin isoforms (Figure 6), but reduced oxidative modification of titin may contribute to this effect of GGA (Bódi et al., 2017). Overall, our findings demonstrate that 4 weeks of oral GGA treatment reduces cardiomyocyte stiffness through both phosphorylation‐dependent and phosphorylation‐independent mechanisms.

### 4.4 Study limitations

In the present study, we did not observe a higher LV stiffness constant β in vehicle‐treated obese ZSF1 rats, in contrast to previous reports (Hamdani et al., 2013; van Dijk et al., 2016) but is consistent with the milder in vivo phenotype exhibited by the obese ZSF1 rats in our study. However, it should be noted that during hemodynamic evaluation prior to sacrifice, vehicle‐treated obese ZSF1 rats were operating at low LVEDP (7.0 ± 1.8 mmHg), and inferior vena cava occlusions starting from this low LVEDP in the flat portion of the EDPVR curve. In the clinical setting, patients with cardiometabolic syndrome and HFpEF often show normal LV function at rest. Notably, during exercise, these patients may experience rapid elevations in LV and pulmonary pressures along with dyspnea as a result of underlying myocardial stiffness. Given that GGA has been found to reduce titin‐based cardiomyocyte stiffness in the present study, it is conceivable that during bouts of elevated preload (such as during exercise or a fluid challenge) the heart's compliance would be less compromised, and hence attenuate the rapid increases in LV filling pressure. However, fluid loading induces sarcomere stretch and affects subsequent cardiomyocyte stiffness measurements, as well as coverage of titin by small HSPs (Franssen et al., 2017; Kötter et al., 2014). Therefore, we opted to refrain from fluid loading during the hemodynamic assessment of LV function. Moving forward, we will conduct studies to evaluate if GGA improves myocardial function in response to a cardiac challenge in cardiometabolic syndrome.

Furthermore, although our data show that GGA attenuates the progression of diastolic dysfunction in obese ZSF1 rats (Figure 1), we did not find any significant effects of GGA on blood pressure, body weight or metabolism in these animals. Focusing on direct effects of GGA on cardiomyocyte stiffness, we have not evaluated potential effects of GGA on the peripheral vasculature. Peripheral microvascular function predicts worsening of HFpEF (Akiyama et al., 2012) and can be improved by GGA (Fujimura et al., 2012), and we will evaluate this effect in HFpEF in future studies.

Finally, we studied male rats exclusively to investigate cardiac and cellular function in a model of the cardiometabolic syndrome that is known to progress to HFpEF. Although HFpEF is more prevalent in women, we aimed to eliminate the influence of sex differences that could impact the quality and interpretation of our analysis. Because we used recombinant HSPB1 and HSPB5 in our cardiomyocyte experiments, we are confident that any possible sex differences that can limit the interpretation of our results is low. These results provide proof‐of‐concept evidence for GGA's downstream effects on cardiomyocyte function.

### 4.5 Conclusions

Our study investigated the potential therapeutic effects of oral treatment with GGA in a model of the cardiometabolic syndrome. Our findings suggest that GGA treatment can increase HSPB1 and HSPB5 to the myofilaments of cardiomyocytes, and reduce cardiomyocyte stiffness by phosphorylation‐dependent and phosphorylation‐independent mechanisms. These effects may delay the development of impaired LV relaxation in the cardiometabolic syndrome. Taken together, our findings suggest that oral GGA treatment could improve therapy in early HFpEF.

## AUTHOR CONTRIBUTIONS

**Conceived and designed the study:** Mark T. Waddingham, Vasco Sequeira, Diederik W. D. Kuster, M. Louis Handoko, Coen A. Ottenheijm, Jolanda van der Velden, Walter J. Paulus, and Etto C. Eringa. **Data collection:** Mark T. Waddingham, Vasco Sequeira, Elisa Dal Canto, Denielli da Silva Gonçalves Bós, Shengyi Shen, and Robbert J. van der Pijl. **Performed data analysis:** Mark T. Waddingham, Vasco Sequeira, Elisa Dal Canto, Denielli da Silva Gonçalves Bós, Shengyi Shen, Robbert J. van der Pijl, and Etto C. Eringa. **Data Interpretation:** Mark T. Waddingham, Vasco Sequeira, Diederik W. D. Kuster, Elisa Dal Canto, M. Louis Handoko, Frances S. de Man, Coen A. Ottenheijm, Jolanda van der Velden, Walter J. Paulus, and Etto C. Eringa. **Drafted the manuscript:** Mark T. Waddingham, Vasco Sequeira, Walter J. Paulus, and Etto C. Eringa. **Read and approved the final manuscript:** Mark T. Waddingham, Vasco Sequeira, Diederik W. D. Kuster, Elisa Dal Canto, M. Louis Handoko, Frances S. de Man, Denielli da Silva Gonçalves Bós, Coen A. Ottenheijm, Shengyi Shen, Robbert J. van der Pijl, Jolanda van der Velden, Walter J. Paulus, and Etto C. Eringa.

## FUNDING INFORMATION

This study was supported by grants from CardioVasculair Onderzoek Nederland (CVON), Dutch Heart Foundation, The Hague, The Netherlands (RECONNECT, EARLY‐HFpEF). Dr. Waddingham was a recipient of a Young Talent Program “Proof‐of‐Concept” Award from the CVON‐RECONNECT consortium of the Dutch Heart Foundation and is currently supported by a JSPS KAKENHI (Grant no. 22K15368).

## CONFLICT OF INTEREST STATEMENT

M.L.H. received an educational/speaker/consultancy fees from Novartis, Boehringer Ingelheim, Daiichi Sankyo, Vifor Pharma, AstraZeneca, Bayer, MSD, and Quin. The remaining authors have no disclosures to report.

## ETHICS STATEMENT

All experiments involving animals were conducted in accordance with the *Dutch Animal Testing Act*, the *European Convention for the Protection of Vertebrate Animals used for Experimental and Other Scientific Purposes (ETS 123)* and the *European Directive for the Protection of Animals used for Scientific Purposes (2010/63/EU)*. A license to conduct animal experiments was obtained from the Central Commission for Animal Experimentation (The Hague, The Netherlands; CCD № AVD114002016768) and the experiments approved by the Institutional Animal Care and Use Committee of the VU University Medical Center, Amsterdam, The Netherlands (Protocol № 768‐FYS17‐01A1). Reporting of animal studies in this paper comply with the ARRIVE guidelines for animal research.
